# Gene Therapy in a Large Animal Model of PDE6A-Retinitis Pigmentosa

**DOI:** 10.3389/fnins.2017.00342

**Published:** 2017-06-20

**Authors:** Freya M. Mowat, Laurence M. Occelli, Joshua T. Bartoe, Kristen J. Gervais, Ashlee R. Bruewer, Janice Querubin, Astra Dinculescu, Sanford L. Boye, William W. Hauswirth, Simon M. Petersen-Jones

**Affiliations:** ^1^Department of Small Animal Clinical Sciences, College of Veterinary Medicine, Michigan State University East Lansing, MI, United States; ^2^Department of Clinical Sciences, College of Veterinary Medicine, North Carolina State University Raleigh, NC, United States; ^3^Department of Ophthalmology, University of Florida College of Medicine Gainesville, FL, United States

**Keywords:** retinitis pigmentosa, Pde6 mutation, gene therapy, canine model, adeno-associated virus

## Abstract

Despite mutations in the rod phosphodiesterase 6-alpha (*PDE6A*) gene being well-recognized as a cause of human retinitis pigmentosa, no definitive treatments have been developed to treat this blinding disease. We performed a trial of retinal gene augmentation in the *Pde6a* mutant dog using *Pde6a* delivery by capsid-mutant adeno-associated virus serotype 8, previously shown to have a rapid onset of transgene expression in the canine retina. Subretinal injections were performed in 10 dogs at 29–44 days of age, and electroretinography and vision testing were performed to assess functional outcome. Retinal structure was assessed using color fundus photography, spectral domain optical coherence tomography, and histology. Immunohistochemistry was performed to examine transgene expression and expression of other retinal genes. Treatment resulted in improvement in dim light vision and evidence of rod function on electroretinographic examination. Photoreceptor layer thickness in the treated area was preserved compared with the contralateral control vector treated or uninjected eye. Improved rod and cone photoreceptor survival, rhodopsin localization, cyclic GMP levels and bipolar cell dendrite distribution was observed in treated areas. Some adverse effects including foci of retinal separation, foci of retinal degeneration and rosette formation were identified in both *AAV-Pde6a* and control vector injected regions. This is the first description of successful gene augmentation for Pde6a retinitis pigmentosa in a large animal model. Further studies will be necessary to optimize visual outcomes and minimize complications before translation to human studies.

## Introduction

Retinitis pigmentosa (RP) is a major cause of heritable vision loss affecting ~1 in 4,000 people (Hartong et al., 2006). It is a genetically heterogenous group of conditions; at the time of writing 67 mapped genes and loci for non-syndromic RP were listed on the RetNet website (the Retinal Information Network https://sph.uth.edu/retnet/home.htm). In many forms of RP the mutated genes are specific to rod photoreceptors leading to rod degeneration and depending on the function of the gene product, a period of abnormal, or absent, rod function may precede rod death. Although cone photoreceptors in many forms of RP do not express the mutant gene, cone degeneration will occur as a consequence of rod degeneration. Initial symptoms of RP result from reduced, or absence of rod function resulting in night blindness. With progression an impairment of cone mediated vision develops leading to visual field constriction and in many cases, complete blindness. Mutations of the alpha or beta subunits of the rod phosphodiesterase (PDE6) result in autosomal recessive RP (arRP). *PDE6-alpha (PDE6A;* OMIM 180071) and *PDE6-beta (PDE6B;* OMIM 180072*)* mutations each account for ~4% of cases of arRP (McLaughlin et al., 1995; Dryja et al., 1999).

The PDE6 complex in rod photoreceptors is composed of two active subunits, alpha and beta, and a pair of gamma inhibitory subunits. Removal of the gamma inhibitory subunits during phototransduction by the activity of transducin, allows the alpha and beta dimer to hydrolyze many cyclic GMP (cGMP) molecules, causing gated channels to close and plasma membrane hyperpolarization to occur (Farber, 1995). Animals lacking functional alpha or beta PDE6 subunit show a reduction in the phototransduction-induced hydrolysis of cGMP resulting in elevated cGMP levels, unregulated influx of calcium, primary rod cell death, and subsequent secondary cone loss (Wensel et al., 2016).

A variety of mouse models for *PDE6B* arRP have been widely used to study disease mechanisms and treatment, including *Pde6b*^*rd*1/*rd*1^ which has a null mutation, and peak rod loss at day 14, (Keeler, 1966; Pittler et al., 1993; Hackam et al., 2004) and the milder *Pde6b*^*rd*10/*rd*10^ missense mutation in which rod loss peaks at day 25 (Gargini et al., 2007; Barhoum et al., 2008). The *Pde6b*^*rd*10/*rd*10^ mouse retina initially contains low levels of Pde6b and retains a rod-mediated electroretinogram response prior to degeneration (Chang et al., 2007). Additional mutagenesis models have been described for *Pde6b* in the mouse, exhibiting a variety of severities of retinal disease (Hart et al., 2005; Chang et al., 2007). Irish setter dogs with a null mutation in *Pde6b* provide a large animal model for *PDE6B* arRP (Farber et al., 1992; Suber et al., 1993). Affected dogs lack Pde6b activity and have elevated retinal cGMP levels arresting normal photoreceptor development from day 13, resulting in retinal degeneration beginning at day 25; rod loss is followed by a progressive loss of cones leading to blindness (Aguirre et al., 1978; Farber et al., 1992). Successful gene therapy studies were initially described for the milder phenotypes of the *Pde6b*^*rd*10/*rd*10^ mouse (Pang et al., 2008, 2011) and *Pde6b*^*H*620*Q*^ hypomorphic mouse model (Davis et al., 2008; Tosi et al., 2011). The early onset, rapid degeneration of the *Pde6b*^*rd*1/*rd*1^ mouse proved to be more challenging to treat with gene therapy, with only partial rescue of the phenotype described (Bennett et al., 1996; Jomary et al., 1997; Kumar-Singh and Farber, 1998; Takahashi et al., 1999; Guo et al., 2015). A recent description of an additional mutation in *Gpr179* in the *Pde6b*^*rd*1/*rd*1^ mouse line may explain some of the difficulty in rescuing the *Pde6b*^*rd*1/*rd*1^ phenotype (Nishiguchi et al., 2015). Following breeding to remove the co-inherited *Gpr179* mutation, gene therapy rescued the *Pde6b*^*rd*1/*rd*1^ phenotype. Successful and sustained rescue of the *Pde6b* mutant dog phenotype with adeno-associated viral vectors encoding canine *Pde6b* when administered by subretinal injection at 20 days of age has been described (Petit et al., 2012; Pichard et al., 2016).

More recently, several mouse models for *PDE6A* arRP have been developed. The severity and rapidity of retinal degeneration of each model is dependent on the gene mutation, varying from slow to rapid (Sakamoto et al., 2009; Sothilingam et al., 2015). We previously described a recessively inherited *Pde6a* null mutation (one base-pair deletion at codon 616 resulting in a frameshift and premature stop codon) in the Cardigan Welsh Corgi breed of dog causing an early-onset and rapid rod and subsequent cone degeneration (Petersen-Jones et al., 1999; Tuntivanich et al., 2009). Outer nuclear layer cell death was present from post-natal day 21 and peaked at post-natal day 27. Cell loss is not related to caspase 3 activity in mouse (Sothilingam et al., 2015) and dog (Tuntivanich et al., 2009) models, indicating a non-caspase dependent mechanism of cell death. *Pde6a* mouse models have reduced amounts of retinal Pde6b in parallel with the decrease in Pde6a (Sakamoto et al., 2009) and the retina of *Pde6a* mutant dogs also lacks beta and gamma subunits of Pde6 indicating a requirement for Pde6a for the normal production or maintenance of the other subunits in the rod outer segment (Petersen-Jones et al., 1999; Tuntivanich et al., 2009). This is in contrast to animal models with *Pde6b* null mutations in which expression of the other *Pde6* subunits is retained (Pittler and Baehr, 1991; Suber et al., 1993). Therapeutic trials using the *Pde6a*^*nmf*363^ mouse model have been reported (Wert et al., 2013, 2014). This particular mouse model has one of the less severe phenotypes. Therapy in this model was successful when performed both prior to photoreceptor loss (Wert et al., 2013) but also when ~50% of photoreceptors had degenerated (Wert et al., 2014). Our initial attempts to rescue the *Pde6a* mutant dog with AAV serotype 5 gene augmentation therapy were unsuccessful (Petersen-Jones, unpublished findings). We hypothesized that because of the severity of the model, transgene expression would be required at a very early age, requiring delivery of treatment to the retina at a stage at which it is still developing and that we would need highly efficient, rapid-onset, viral vectors. A high efficiency adeno-associated virus serotype-8 with capsid mutation was successful in rescuing the rapid degeneration seen in the *Aipl1* mutant mouse (Ku et al., 2015) and the *Pde6a*^*nmf*363^ mouse model, (Wert et al., 2013) even when delivered at more advanced stages of disease (Wert et al., 2014). We have shown that tyrosine capsid-mutant AAV8 viral vectors have an onset of gene expression within 2–3 days in the canine retina, (Mowat et al., 2014) and therefore show promise for the rescue of the rapid degeneration seen in the *Pde6a* mutant dog.

Currently there are no definitive treatments for RP associated with retinal PDE6 mutations. Recent phase I/II clinical trials have demonstrated the safety and efficacy of gene augmentation using adeno-associated viral vectors in patients with Leber Congenital Amaurosis due to *RPE65* gene mutations (Bainbridge et al., 2008; Cideciyan et al., 2008, 2009a,b; Hauswirth et al., 2008; Maguire et al., 2008, 2009; Simonelli et al., 2010). Experimental efficacy in *Pde6b* models will allow translation into human clinical trials in the near future, however only limited preclinical trials have been performed in PDE6A models.

In this publication, we present the results of tyrosine capsid-mutant AAV8 *Pde6a* gene therapy delivered by subretinal injection to young *Pde6a* mutant dogs. We show that treatment results in partial return of visual function as determined by electroretinography and objective vision assessment. The treatment effect is sustained for at least 4 months, and preserves photoreceptors in the treated area. Treatment reverses retinal cGMP accumulation, reduces mislocalization of rhodopsin, limits glial activation, and maintains dendritic arbors of rod bipolar cells. However, within the treated areas adverse treatment related effects were seen. These include persistent regions of retinal separation and rosette formation.

## Materials and methods

### Subjects

Animals were bred and housed in a facility at Michigan State University. Animals (*n* = 10) were all homozygous for a mutation in *Pde6a* (Petersen-Jones et al., 1999; Tuntivanich et al., 2009). Animals were housed on a 12-h light: dark cycle, and care was in compliance with the ARVO Statement for the Use of Animals in Ophthalmic and Visual Research, with all procedures being performed with approval from the Michigan State University Institutional Animal Care and Use Committee.

### AAV constructs

Recombinant AAV vectors were manufactured and purified by previously described methods, (Hauswirth et al., 2000; Zolotukhin et al., 2002; Zhong et al., 2008; Pang et al., 2011; Petrs-Silva et al., 2011) including purification and concentration by column chromatography. Vector titer was determined by real-time PCR and aliquots were resuspended in balanced salt solution (BSS, Alcon Laboratories, Fort Worth, TX, USA) containing 0.014% tween 20 (Sigma Aldrich, St Louis MO). The vector used was an AAV8 capsid with a single tyrosine to phenylalanine mutation at position 733 (Petrs-Silva et al., 2011) containing a construct expressing either canine *Pde6a* cDNA (*AAV-Pde6a*) or humanized green fluorescent protein cDNA (*AAV-GFP*) driven by the ubiquitous truncated chimeric CMV-chicken beta-actin promoter (smCBA).

### Subretinal injections

All vectors were diluted to 4.19 × 10^11^ viral genomes/ml using sterile balanced salt solution (BSS) before injection. Under general anesthesia, 50–100 μl of vector preparation was delivered by subretinal injection as previously described (Petersen-Jones et al., 2009; Mowat et al., 2014). Injections were placed in the superior tapetal portion of the retina, close to the nasal, or temporal aspect of the optic nerve head. Immediately after subretinal injection, all dogs received a subconjunctival injection of 2 mg dexamethasone solution and 2 mg of methylprednisolone acetate. Dogs were injected at a mean of 36 days of age (range 29–44 days, Table 1). One eye from each dog was injected with *AAV-Pde6a*, the contralateral eye was uninjected (*n* = 5) or injected with *AAV-GFP* (*n* = 5).

**Table 1:** Details of dogs used for experimental procedures.

**Animal number**	**Age at injection (days)**	**Injection OD**	**Injection OS**	***In vivo* data collection**	**Age at euthanasia (days)**
10-023	30	*AAV2/8mut733.CBAp.cPDE6a*	None	ERG	92
				VT: ob	
				FI	
11-001	29	*AAV2/8mut733.CBAp.cPDE6a*	None	ERG	125
				VT: ob	
				FI	
11-006	29	*AAV2/8mut733.CBAp.cPDE6a*	None	ERG	125
				VT: ob	
				FI	
11-007	29	*AAV2/8mut733.CBAp.cPDE6a*	None	ERG	125
				VT: ob	
				FI	
11-008	29	*AAV2/8mut733.CBAp.cPDE6a*	None	ERG	125
				VT: ob	
				FI	
12-046	42	*AAV2/8mut733.CBAp.cPDE6a*	*AAV2/8mut733.CBAp.GFP*	ERG	208
				VT: ob/cd	
				OCT	
				FI	
12-047	41	*AAV2/8mut733.CBAp.cPDE6a*	*AAV2/8mut733.CBAp.GFP*	ERG	208
				VT: ob/cd	
				OCT	
				FI	
12-048	41	*AAV2/8mut733.CBAp.cPDE6a*	*AAV2/8mut733.CBAp.GFP*	ERG	208
				VT: ob/cd	
				OCT	
				FI	
12-060	44	*AAV2/8mut733.CBAp.cPDE6a*	*AAV2/8mut733.CBAp.GFP*	FI	58
12-061	44	*AAV2/8mut733.CBAp.cPDE6a*	*AAV2/8mut733.CBAp.GFP*	ERG, FI	72

*OD, Right eye; OS, left eye; ERG, electroretinography; VT, vision testing; ob, obstacle course; cd, 4 tunnel choice device; FI, fundus imaging; OCT, optical coherence tomography imaging*.

### Ophthalmic evaluation and fundic imaging

Slit-lamp biomicroscopy (SL15, Kowa, Japan), binocular indirect ophthalmoscopy (Welch Allyn, Skaneateles Falls, NY, USA) and wide-angle color and fluorescent digital fundus imaging (RetCam II, Clarity Medical Systems, Pleasanton CA) were performed immediately following injection and then at regular intervals.

### Confocal scanning laser ophthalmoscopy and spectral domain optical coherence tomography

Confocal scanning laser ophthalmoscopy (cSLO) and spectral domain optical coherence tomography (SD-OCT) imaging (Spectralis HRA+OCT, Heidelberg Engineering, Carlsbad CA USA) was performed under general anesthesia. Pupils were dilated with tropicamide (Tropicamide Ophthalmic Solution UPS 1%; Falcon Pharmaceuticals Ltd., Fort Worth, TX, USA), and the eyes were maintained in primary gaze for imaging. Wide-field fundus images were obtained by cSLO using infrared imaging (820 nm laser) and fluorescent imaging (488 nm laser). Retinal cross-section images were obtained using SD-OCT with an 820 nm wavelength laser and a 30° lens. The injected area was imaged in both the *AAV-Pde6a* treated and *AAV-GFP* treated eye (*n* = 3 dogs). Images were obtained at 1, 3, and 5 months following injection. Quantification of OCT segment thickness was performed using the integrated Heidelberg Eye Explorer (Heyex) software. The thickness of the outer nuclear layer was measured in six regions throughout the injected area, and a mean value was calculated per eye, per timepoint to compare statistically.

### Electroretinography

Bilateral electroretinography (ERG) was performed at 0.5, 1, 3, and 4 months following injection, using methods previously described (Annear et al., 2011). The amplitude of the peak scotopic threshold response (STR; detected at a flash luminance below that needed to elicit a b-wave) at each timepoint was calculated and used in analysis.

### Vision testing

Subjective assessment of visual ability was used in a subset of animals (see Table 1). This was performed using an obstacle course, through which the animals navigated using either the *AAV-Pde6a* treated eye or the control eye. Objective assessment of a subset of animals was performed using a previously described four-choice device, (Gearhart et al., 2008) allowing quantifiable testing of visual ability at a range of light intensities. Light intensities were adjusted to range from a dim light that tests rod only vision through mesopic to photopic levels (full room light).

### Tissue processing and sectioning

Animals were euthanized using barbiturate overdose (Fatal Plus, Vortech Pharmaceuticals, Dearborn, MI, USA) 0.5–6 months following injection (Table 1). Eyes were prepared for sectioning as previously described (Mowat et al., 2013, 2014). Globes were sectioned sagitally through the vertical meridian. Fourteen micron frozen sections were taken from the area including the optic nerve and of the nasal and temporal retina, to encompass both injected and uninjected areas within the same eye.

### Immunohistochemistry

Reagents utilized, and basic immunohistochemistry (IHC) protocols performed were as previously described (Mowat et al., 2014). Specific antibodies and conditions utilized in this study are outlined in Table 2.

**Table 2:** Primary antibodies used for immunohistochemistry.

**Antigen target**	**Antibody details and working dilution**	**Manufacturer**	**Secondary antibody and concentration (all from Invitrogen Corp. Carlsbad CA, USA)**
PDE6 (all isoforms)	Rabbit polyclonal 1:5000	Cytosignal, Irvine, CA, USA	AlexaFluor anti-rabbit 546 1:500
PDE6B	Rabbit polyclonal 1:250	Novus Biologicals, Littleton, CO, USA	AlexaFluor anti-rabbit 546 1:500
Rhodopsin	Mouse monoclonal 1:10	Labvision (Thermoscientific), Kalamazoo MI, USA	AlexaFluor anti-mouse 488 1:500
Cone arrestin (LUMif) (Li et al., 2003)	Rabbit polyclonal 1:10,000	Kind gift of Cheryl M. Craft, University of Southern California	AlexaFluor anti-rabbit 546 1:500
Protein kinase-C alpha	Mouse monoclonal 1:500	BD Biosciences, San Diego, CA, USA	AlexaFluor anti-mouse 488 1:500
Glial Fibrillary Acidic Protein	Rabbit polyclonal 1:500	Dako North America, Carpinteria, CA, USA	AlexaFluor anti-rabbit 546 1:500
Cyclic GMP	Sheep formaldehyde conjugated 1:3000	Gift of Dr. HWM Steinbusch, Maastricht University Medical Center, Maastricht, NL	AlexaFluor anti-sheep 488, 1:500

### Imaging and quantification of immunolabeled retinal sections

Slides were imaged using a fluorescence microscope (Nikon Eclipse 80i, Nikon instruments Inc., Melville, NY, USA) using commercial image capture software (MetaVue, Molecular Devices, Sunnydale, CA, USA). For rod and cone counts, slides co-labeled for Pde6 and cones (with human cone arrestin antibody) were imaged. Within each section, three images were taken from the superior area (in injected eyes, defined by positive PDE6 IHC or GFP expression). In control eyes, the region imaged was defined by the site of injection of the *AAV-Pde6a* treated eye. Three sections were imaged, giving 9 regions per eye. A mean value was calculated for cone and rod number in a 500 μm section of retina was calculated as previously described (Mowat et al., 2013). Representative images of immunohistochemistry were also captured using a confocal microscope (Olympus FluoView 1000, Center Valley, CA).

### Statistical analysis

Statistical analysis was performed using GraphPad Prism 5.0a (GraphPad Software Inc, La Jolla, CA, USA). For vision testing data, a two-way analysis of variance was used to assess the effect of treatment and time to exit the device or light intensity on the variable examined. A Bonferroni post-test correction was used to compare replicate means. For rod and cone counts comparing treated eyes with contralateral untreated or GFP treated eyes, a two-tailed paired student's *t*-test was performed. Effects were considered significant if *P* < 0.05. In all figures, graphs represent mean ± standard error of the mean.

## Results

### *Pde6a* gene augmentation results in a scotopic threshold response in the ERG and improved dim light vision

**Figure 1:**
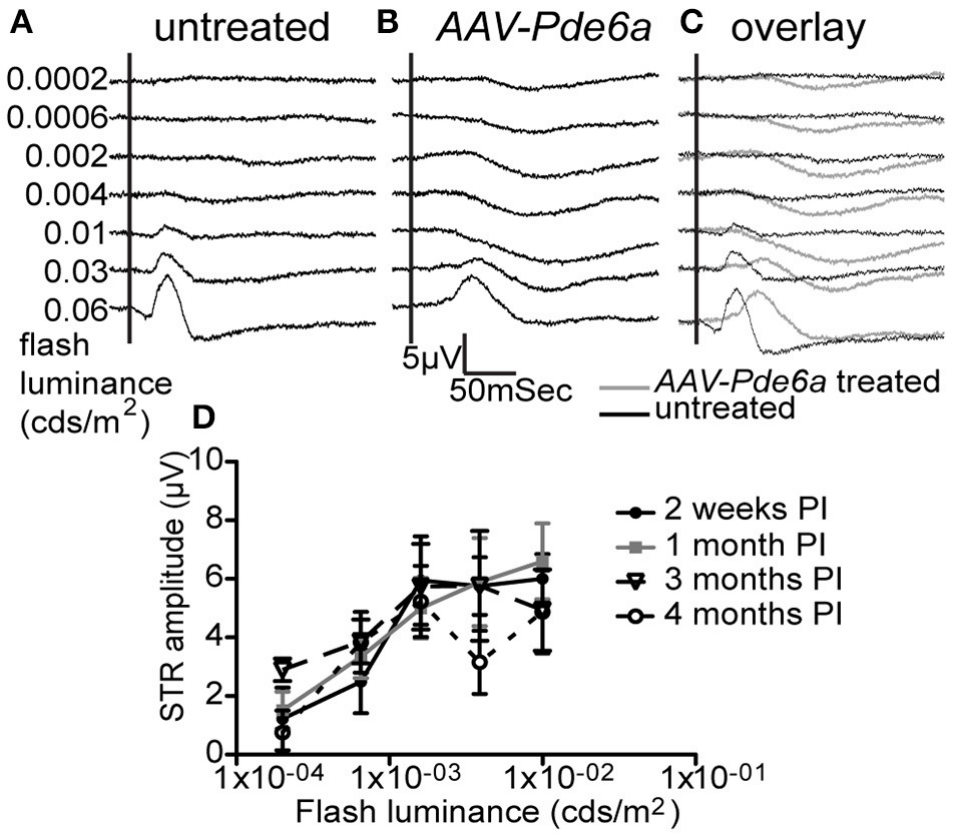
Electroretinography. Five of 9 *AAV-Pde6a* treated eyes had a measureable scotopic threshold response (STR) on ERGs at light intensities below b-wave threshold (representative traces shown in **A–C**). No untreated eye or AAV-GFP injected eye had the presence of an STR. The STR in those eyes with a detectable response increased in amplitude with increasing flash luminance, and the amplitude was sustained throughout the treatment period **(D)**.

Untreated and control *AAV8-GFP* injected eyes of *Pde6a* mutant dogs did not have detectable rod ERG responses. There were no scotopic threshold responses, and the dark- and light-adapted b-wave thresholds were similar and apart from the lowest stimulus intensities the b-wave amplitudes were similar in amplitude (Figure 1A). At 1 month following subretinal injection with *AAV-Pde6a* evidence of rod-mediated ERG responses were recordable in 5 out of the 9 dogs (one dog was terminated prior to the 1 month ERG). In each of the five dogs with a detectable ERG difference the main effect of therapy was the introduction of a scotopic threshold response (STR) which is a rod driven response (Figures 1A–C) (Sieving et al., 1986). The STR response was sustained to 4 months after treatment (Figure 1D). The same dogs had a dark-adapted ERG b-wave in response to flashes just above threshold with a longer implicit time than in controls. The longer implicit time is more in keeping with a rod rather than cone response, although the dark-adapted b-wave threshold and amplitude was not improved by therapy.

**Figure 2:**
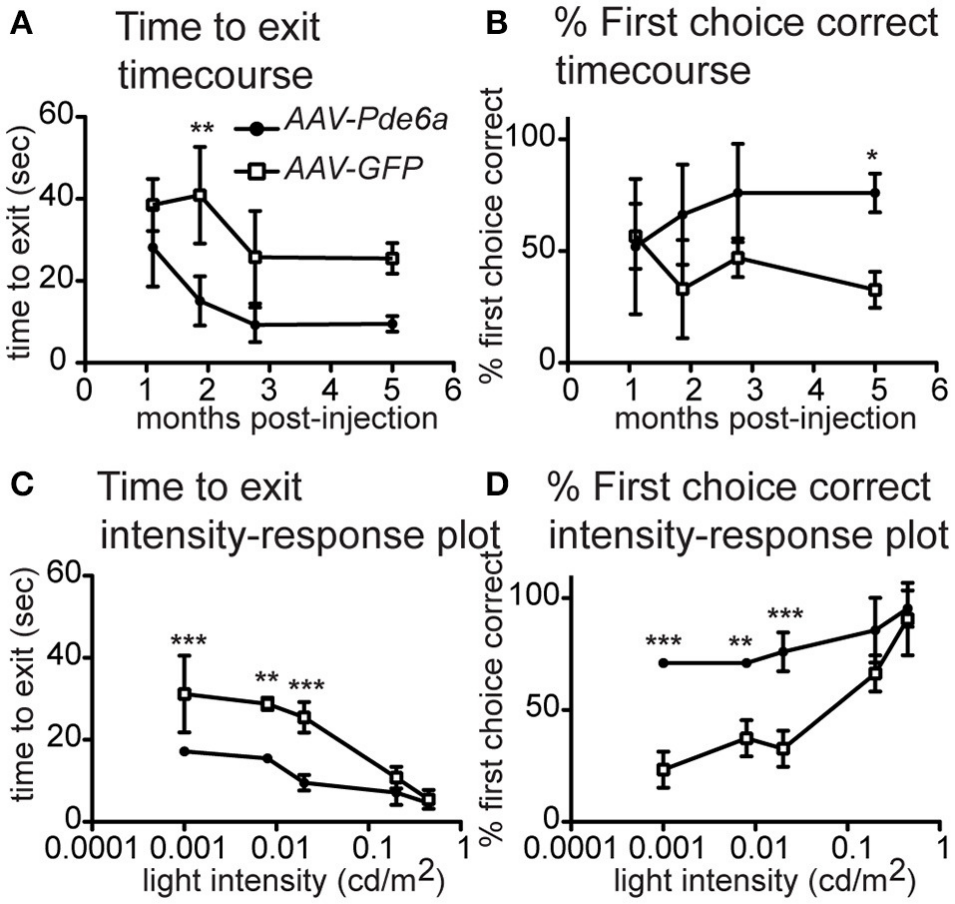
Vision Testing. The visual navigation ability of a subset of treated dogs (*n* = 3) was tested sequentially after injection (*AAV-Pde6a* and contralateral *AAV-GFP)*. At mesopic lighting intensity (0.02 cd/m2), dogs tended to navigate more quickly using the *AAV-Pde6a* treated eye **(A)**, and make fewer incorrect choices **(B)**, although using a two-way repeated measures ANOVA, only select time-points were significantly different from control injected eyes. Consequently, even lower light intensities were tested at only the 5 month post-injection time-point. Following treatment, animals navigated significantly more quickly using *AAV-Pde6a* treated eyes **(C)** and made more correct exit choices than contralateral *AAV-GFP* treated eyes **(D)** at the three lowest light intensities tested (0.02, 0.008–0.01, 0.001–0.003 cd/m^2^). (^*^p < 0.05, ^**^p < 0.01, ^***^p < 0.001, two way ANOVA using treatment and either time or light intensity as the two independent variables, Bonferroni post-test).

Dogs with recordable STR responses also showed an improvement in dim light visual function in the *AAV-Pde6a* treated eye, confirming the ERG findings of improved rod function (Figure 2 and Supplementary Video 1). At a low light intensity (0.02 cd/m^2^), animals tended to navigate more rapidly through the device using the *AAV-Pde6a* treated eye (Figure 2A) and made fewer incorrect first choice of exit tunnel (Figure 2B). Overall eyes treated with the therapeutic vector *(AAV-Pde6a)* had significantly improved visual function compared to the control eyes (AAV-GFP) (*p* < 0.01 ANOVA), although statistical significance was only achieved at certain time-points following injection. To further investigate rod-mediated vision, at 5 months following injection, a range of extremely low light levels were tested (range 0.02–0.003 cd/m^2^), and animals navigated significantly more quickly (Figure 2C) and with fewer mistakes (Figure 2D) using *AAV-Pde6a* treated eyes compared with *AAV-GFP* treated eyes. Brighter light (cone-mediated) vision was maintained in both *AAV-Pde6a* and *AAV-GFP* treated eyes.

### Pde6a gene augmentation results in sustained preservation of the retinal photoreceptor nuclear layer

**Figure 3:**
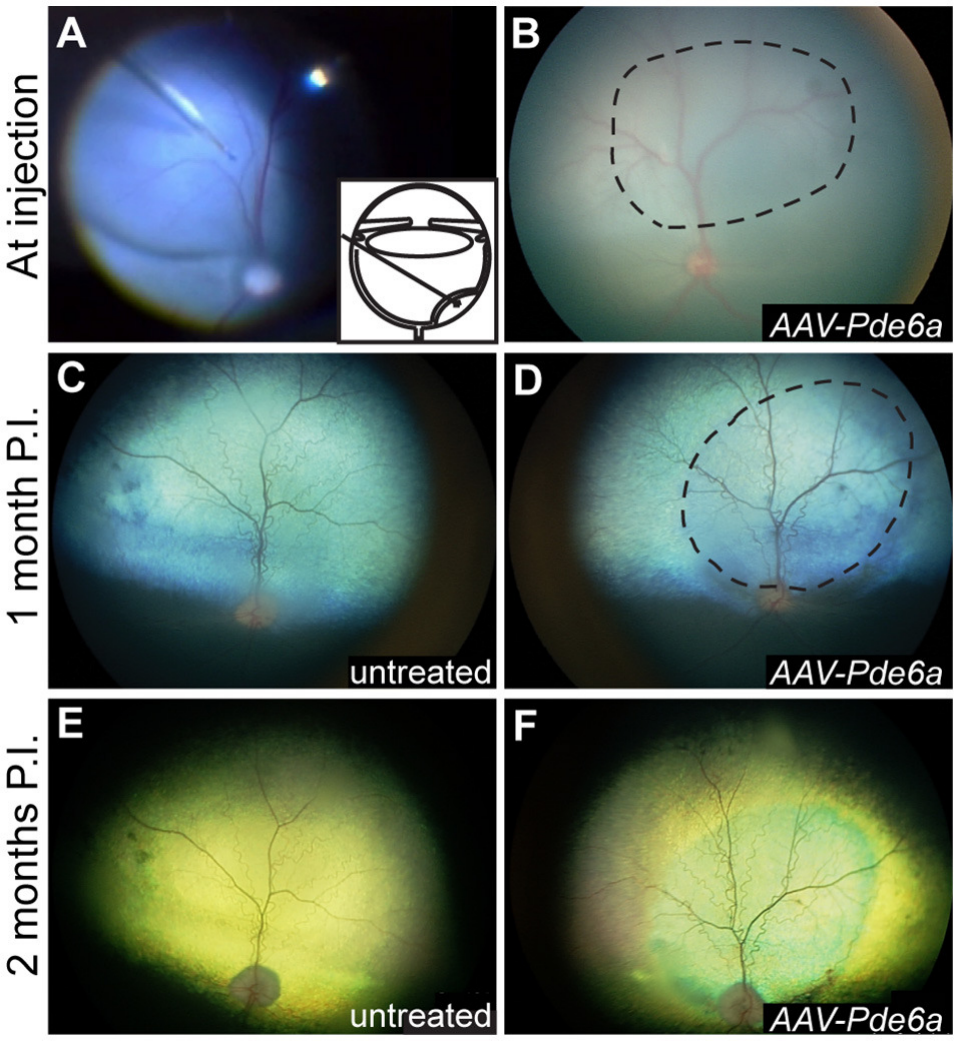
Fundus photography. Subretinal injections of vectors were performed at 29–44 days of age; a representative intraoperative color photograph and schematic of the subretinal injection technique are shown in **(A)**. The subretinal bleb often flattened significantly immediately after injection **(B)**, and was visibly resolved within 24 h in all eyes. One month following injection, mild arteriolar attenuation was present in control, uninjected eyes **(C)**, and mild preservation of normal arteriolar diameter was present in the treated region of *AAV-Pde6a* injected eyes **(D)**. By 2 months following injection untreated eyes and untreated regions of *AAV-Pde6a* treated eyes had significant arteriolar and mild venular attenuation **(E)**, which contrasted with preserved arteriolar and venular vessel diameters in the treated area **(F)**. Dashed line delineates the injected area.

Resolution of the retinal bleb associated with subretinal injection occurred within 24 h in all injected eyes (Figures 3A,B). GFP expression was detected by fluorescent fundus imaging performed 1-month after injection in those eyes treated with *AAV-GFP*, and was sustained (data not shown). By 1 month following injection, mild arteriolar attenuation, which it typical of the *Pde6a* mutant dog natural history, was visible in untreated eyes, and ophthalmoscopic evidence suggestive of preservation of retinal thickness could be detected in the treated region in *AAV-Pde6a* treated eyes (Figures 3C,D). The degree of retinal thinning in the dog can be judged by the development of tapetal hyperreflectivity in the tapetal fundus and the injected regions subjectively were less hyperreflective than the adjacent untreated tapetal fundus. By 2 months following injection, significant arteriolar and mild venular attenuation was visible in untreated eyes (Figure 3E), and untreated regions of *AAV-Pde6a* treated eyes, compared with preservation of more normal vascular thickness in the treated area (Figure 3F).

**Figure 4:**
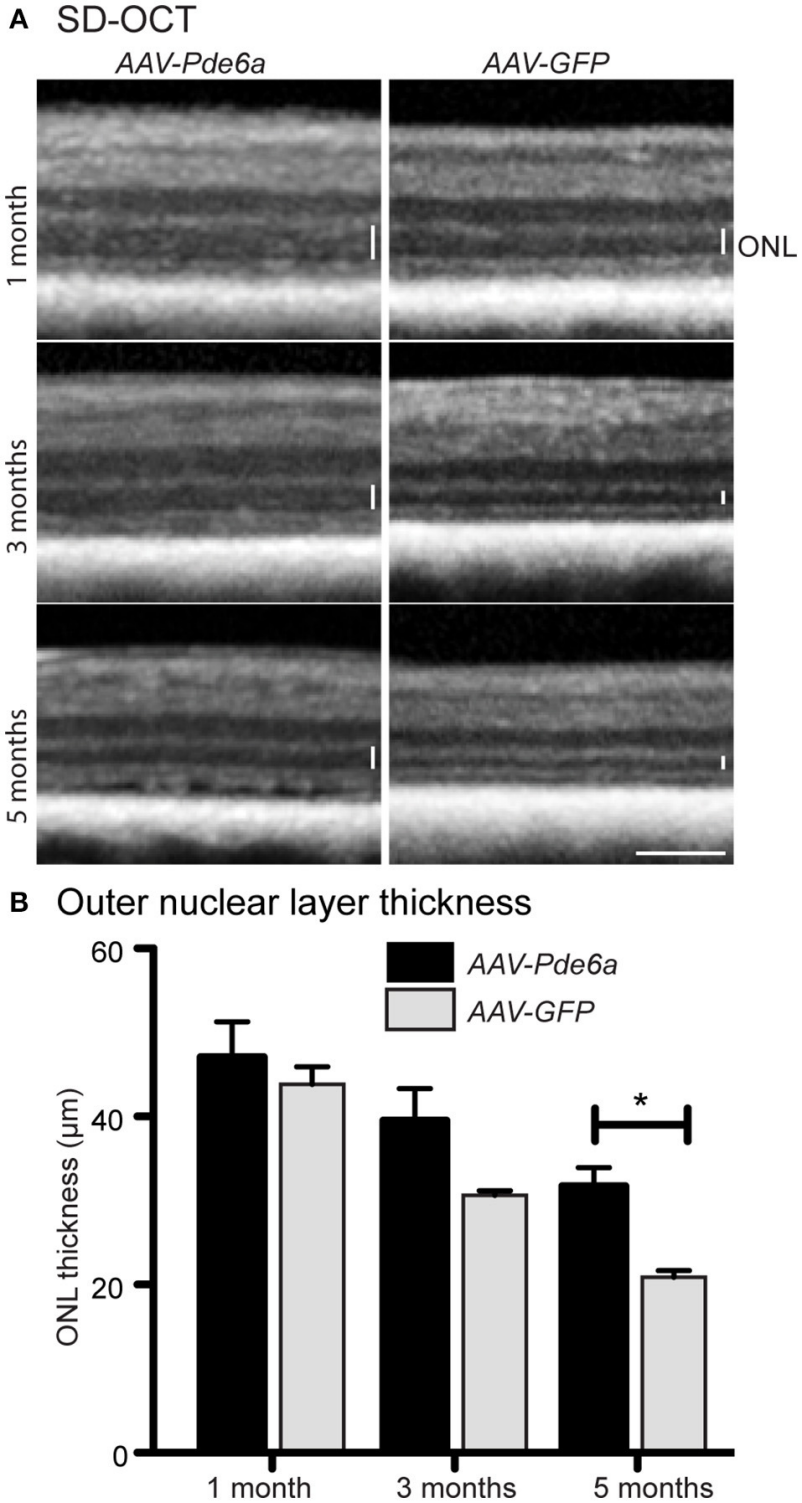
Spectral domain optical coherence tomography. SD-OCT was performed on three animals injected with *AAV-Pde6a* at 41–42 days of age, contralateral eyes were injected with *AAV-GFP*. Animals were imaged at 1, 3, and 5 months post-treatment **(A)**. The outer nuclear layer in *AAV-Pde6a* treated eyes was thicker compared with contralateral *AAV-GFP* treated eyes, and this difference was significant at 5 months post-injection **(B)**. ^*^p < 0.05, two-way repeated measures ANOVA, scale bar represents 200 μm.

SD-OCT was performed in three animals (12-046, 12-047, and 12-048). These animals were treated at 41–42 days of age; the contralateral eye was treated with *AAV-GFP* (Table 1). *AAV-Pde6a* treated eyes had relatively less thinning of the outer nuclear layer (ONL) throughout the 5 months following treatment compared with *AAV-GFP* treated eyes in which thinning of the ONL progressed more rapidly (Figure 4A). By 5 months following injection, the ONL in the *AAV-Pde6a* treated area was significantly thicker than that of the contralateral eye (*AAV-GFP* treated) (Figure 4B). Note that in some of the gene therapy treated eyes retinal abnormalities developed as described further below. Careful assessment of the zones on SD-OCT showed that in *AAV-Pde6a* treated retinal regions there the region that represents the photoreceptor inner and outer segments have improved definition and indicated that the inner/outer segment length was greater than in the adjacent untreated region of the same eye and the in the comparable retinal region of the *AAV-GFP* treated eye (Supplementary Figure 1). Supplementary Figure 1 also shows a heat map of a *AAV-Pde6a* treated retinal region showing photoreceptor preservation compared to the immediately adjacent untreated region of the same eye.

### *AAV-Pde6a* treated retinal regions demonstrate outer segment Pde6 expression and more appropriate localization of rhodopsin to the outer segment

**Figure 5:**
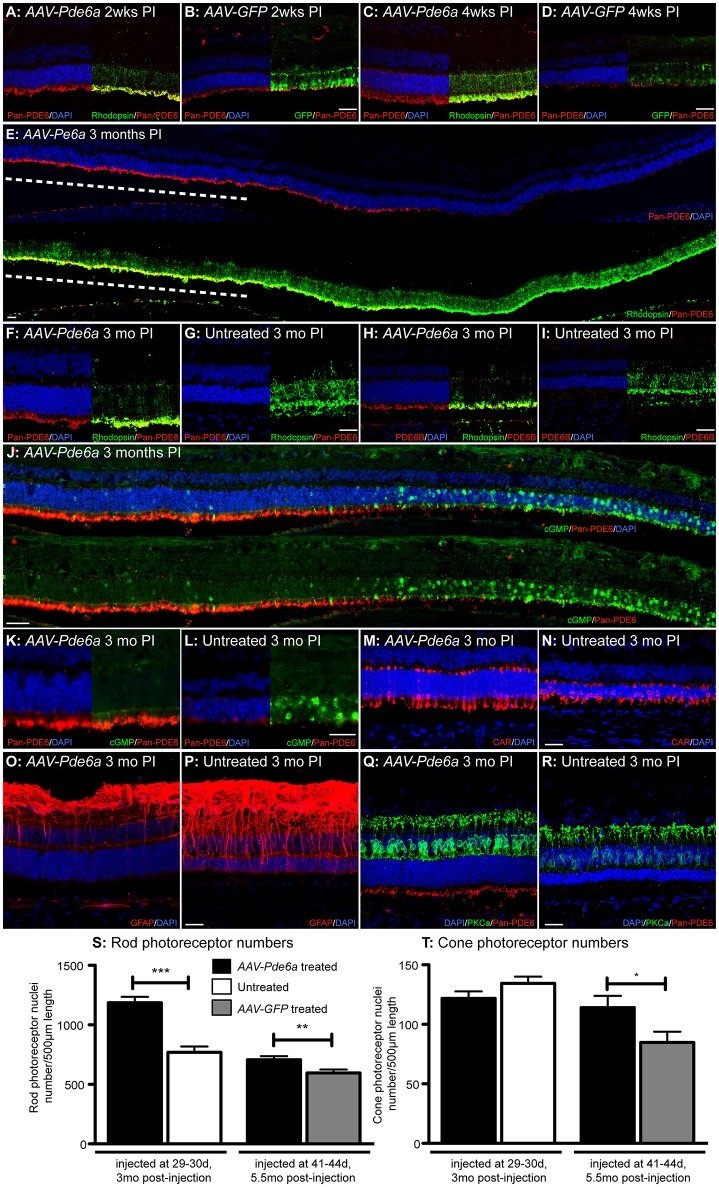
Histological features of *Pde6a* gene augmentation treatment. Two weeks following injection increased immunolabeling for PDE6 protein was detected in the *AAV-Pde6a* treated eye **(A)**, and GFP expression was detected at this stage in the contralateral *AAV-GFP* injected eye **(B)**. PDE6 expression was sustained in the *AAV-Pde6a* treated eye after 4 weeks **(C)** but very little was detected in eyes treated with *AAV-GFP* **(D)**. Three months following treatment with *AAV-Pde6a*, outer segment detection of PDE6 was sustained in the injected area (dashed white line; **E**), and there was reduction in the amount of rhodopsin mislocalized into the rod cell bodies in the treated region **(F)** compared with significant cell body mislocalization in untreated contralateral eyes **(G)**. Pde6b, a subunit not directly delivered by treatment with *AAV-Pde6a*, was also detected in the rod outer segments of *AAV-Pde6a* treated eyes **(H)** compared with minimal signal in untreated contralateral eyes **(I)**. Cyclic GMP levels were reduced in the injected area of eyes treated with *AAV-Pde6a* **(J,K)**, but was detected at much higher levels in outer nuclear cell bodies in untreated regions of the same eyes and also in untreated eyes **(L)**. Cone inner and outer segment morphology was better preserved in *AAV-Pde6a* treated eyes **(M)** compared with untreated eyes **(N)**. GFAP positive fibrils extending into the outer nuclear layer were only detected in a few glia in *AAV-Pde6a* treated eyes **(O)** compared with significant numbers in untreated eyes **(P)**. The rod bipolar dendrites appeared to have a normal distribution in the treated retinal region **(Q)** whereas by this age there was clear dendrite retraction in the untreated retinal regions **(R)**. In both earlier and later treated groups rod photoreceptor nuclei numbers in treated areas of *AAV-Pde6a* injected eyes were significantly higher than matched regions in the contralateral eye **(S)**. Cone numbers were not preserved in eyes treated early and harvested 3 months following injection compared with untreated controls, but there were significantly more cones surviving 5.5months after later treatment with *AAV-Pde6a*, compared with *AAV-GFP* treated controls **(T)**. CAR, cone arrestin; GFAP, glial fibrillary acidic protein; PKCa, protein kinase C-alpha; DAPI, 4′,6-diamidino-2-phenylindole; PI, post-injection. Scale bars in all images represent 25 μm. ^*^p < 0.05, ^**^p < 0.01, ^***^p < 0.001, paired *t*-test. Confocal microscopy images for all except **(J–L)** (standard fluorescence microscopy).

The single animal (12-061) examined at 2 weeks following injection, had obvious Pde6 protein readily detectable by IHC in the photoreceptor outer segments in the treated area of *AAV-Pde6a* injected eye (Figure 5A) compared with a low level of labeling in the *AAV-GFP* treated eye (Figure 5B). GFP expression was detected by IHC at 2 weeks following injection in *AAV-GFP* treated eyes (Figure 5B). In the animal (12-060) examined at 4 weeks a more marked difference in outer segment Pde6 expression was noted between the two eyes (Figures 5C,D), and only small amounts of remaining Pde6 were detected in some of the control injected eyes at this timepoint, Pde6 protein was not detected in control eyes at later timepoints. Pde6 protein expression was sustained for at least 3 months in the *AAV-Pde6a* injected region (Figure 5E). Rhodopsin IHC showed there was less mislocalization to the rod cell body in the *AAV-Pde6a* treated retinal regions and improved trafficking to the outer segments (Figures 5F,G). Pde6b expression was also detected in treated areas, but not in untreated areas (Figures 5H,I), suggesting that treatment helped stabilize the full Pde6 complex as the other subunits are secondarily deficient in this model (Tuntivanich et al., 2009). Very low levels of cyclic GMP (cGMP) were detectable by IHC in the injected area of the *AAV-Pde6a* treated eye, but outside of the injected area, abnormally high levels of cGMP were present in the outer nuclear layer, corresponding with loss of outer nuclear cell layer nuclei and absence of Pde6 expression (Figures 5J–L). Cone photoreceptors in *AAV-Pde6a* treated areas appeared to have a normal morphology compared to those in the untreated areas which were stunted (Figures 5M,N). Glial fibrillary acidic protein (GFAP) expression within the retina was also lower in Pde6 expressing areas (Figure 5O). In untreated areas, GFAP positive filaments more frequently extended to the photoreceptor outer nuclear layer, indicating gliosis (Figure 5P). Rod bipolar cell dendritic arbors were maintained with a normal appearance in the treated retinal regions (Figure 5Q), whereas there was retraction of the dendritic arbors in the untreated retinal regions (Figure 5R).

### *Pde6a* gene augmentation results in prolonged survival of rod and cone photoreceptors

The surviving nuclei of rods (Figure 5S) and cones (Figure 5T) were quantified in the two groups of treated animals (five animals treated at 30 days, contralateral eye untreated; three animals treated at 41–42 days, contralateral eye *AAV-GFP* treated, Table 1). Rod photoreceptor nuclei in the *AAV-Pde6a* treated eye were significantly preserved in both groups, when compared to an analogous region in the contralateral eye (either untreated or *AAV-GFP* treated; Figure 5S). Cone photoreceptor numbers were not different between groups in animals treated at 30 days of age and maintained for 3 months, but cone nuclei were significantly preserved in animals treated with *AAV-Pde6a* at 41–42 days and maintained for 5.5 months, compared with *AAV-GFP* treated controls (Figure 5T). Comparison of rod numbers per unit retinal length were made between the two control groups (untreated and AAV-GFP injected) and there were no significant differences (data not shown).

### There are complications associated with AAV-mediated gene augmentation in Pde6a mutant dogs

**Figure 6:**
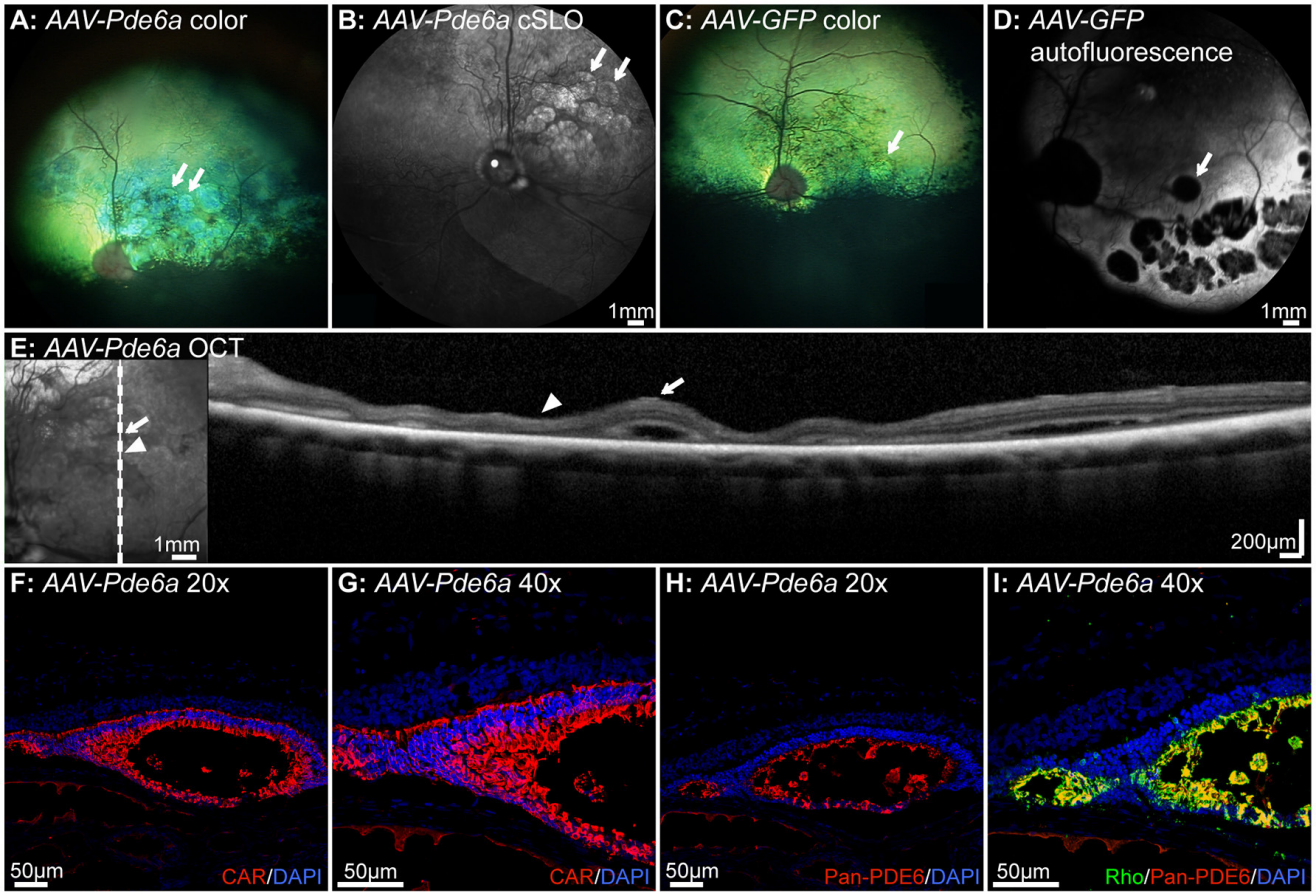
Complications associated with gene therapy administration in *Pde6a* mutant dogs. In the 3 dogs injected at 41–42 days of age and injected bilaterally (one eye with *AAV-Pde6a*, one eye with *AAV-GFP*), changes on fundus images indicating retinal thinning were observed both in eyes injected with *AAV-Pde6a* **(A,B)** and with *AAV-GFP* **(C,D)**. Eyes treated with *AAV-GFP* had a hyperfluorescent area delineating the injected region, and contained within that areas of focal hypofluorescence, possibly indicating loss of GFP fluorescence or cell loss **(D)**. White arrows delineate the corresponding areas of abnormality in **(A–D)**. SD-OCT imaging of these areas **(E)** showed foci of retinal separation (white arrow) interspersed with areas of retinal thinning, predominantly of the outer nuclear layer (white arrowhead). Histologically, the areas of retinal separation appeared to be primitive rosettes, with cone cell bodies lining a lumen **(F,G)** containing cone and rod outer segments expressing transgenic Pde6 **(H)** and endogenous rhodopsin **(I)**. CAR: cone arrestin, DAPI, 4′,6-diamidino-2-phenylindole; Rho, rhodopsin.

Five dogs were injected at 41–44 days of age. Two of these dogs were euthanized at 2 and 4 weeks respectively following injection, and not assessed for retinal thickness. In the remaining three dogs there was evidence of regions of retinal thinning within the treated areas on fundus images in both *AAV-Pde6a* (Figures 6A,B) and *AAV-GFP* treated eyes (Figures 6C,D). cSLO images show changes in *AAV-Pde6a* treated eyes (Figure 6C), and in fundus autofluorescence images of *AAV-GFP* treated eyes, there were multifocal round to oval-shaped regions where fluorescence was diminished within the treated area, most likely due to the absence of cells expressing GFP in degenerate areas (Figure 6D). SD-OCT images showed that these regions represented either focal retinal detachment (Figure 6E, arrow), or marked thinning of the photoreceptor outer nuclear layer (Figure 6E, arrowhead). Histology of affected eyes demonstrated that the detached areas appeared to represent rosettes that contained outer segments of both cones (Figures 6F,G), and rods (Figures 6H,I). The rosettes were present predominantly within injected areas—surviving rod photoreceptors expressed Pde6 in *AAV-Pde6a* treated eyes (Figures 6F,G), and GFP in *AAV-GFP* treated eyes (data not shown).

## Discussion

*AAV-Pde6a* gene augmentation therapy in a small number of young *Pde6a* mutant dogs resulted in restoration of Pde6 protein in the outer segments of rods in all treated retinal areas. Restoration of Pde6 in the outer segments was associated with rod function on ERG in 5 of 9 eyes tested as evidenced by development of a scotopic threshold response and alterations in the timing of the b-wave. These same five treated eyes had improved dim-light vision compared to the control eyes. The scotopic threshold response is a rod mediated response (despite the measured electrical signal originating in the inner retina) and appears at a stimulus luminance close to visual threshold (Sieving et al., 1986; Naarendorp and Sieving, 1991). The slow negative STR waveform initially increases in amplitude with increasing stimulus luminance but becomes obscured by the incursion of the b-wave. Previous studies in models have suggested that the STR threshold tracks remaining functional vision better than the ERG b-wave (Bush et al., 1995) The scotopic b-wave in the eyes that showed rescue had a longer implicit time, more in keeping with a rod-response, although the flash luminance needed to induce an ERG b-wave was not reduced by treatment. Previous ERG studies in dogs have shown that the rod-mediated b-wave is a slower waveform than the contribution of dark-adapted cones to the scotopic responses to stronger stimuli that in normal dogs result in a mixed rod/cone response (Narfström et al., 1995) *Pde6a* mutant dogs that have not been treated, have no evidence of rod ERG responses and exhibit cone-only function (Tuntivanich et al., 2009). The small delayed (compared to control eyes), dark-adapted b-wave in treated eyes reflects a low level of functional rod rescue even allowing for the fact that only a proportion of the retina was treated (blebs typically extended over one-fifth to one-third of the retina). The dark-adapted b-wave amplitudes recorded might also be low because (1) only a proportion of rods in the treated area are functional, or (2) the transduced rods have short outer segments reducing the individual response from each rod, or (3) rod bipolar cell dendritic connections to rods may have been reduced (as reported in other PDE6 models; Strettoi and Pignatelli, 2000), or a combination of these possibilities. The increase in dark-adapted b-wave implicit time combined with the presence of an STR, improved dim light vision, Pde6 localization to outer segments on IHC and photoreceptor structural preservation, provides convincing evidence of *AAV-Pde6a* mediated rod rescue.

Improvement in visual performance is reported to be a more sensitive measure of visual function than ERG. For example, in *RPE65* gene augmentation therapy for Leber Congenital Amaurosis improved visual function can be detected in the absence of an ERG improvement both in human patients in phase I/II clinical trials, but also in the dog model when receiving a low dose of therapeutic vector (Bainbridge et al., 2008, 2015; Hauswirth et al., 2008; Maguire et al., 2008). This difference between the sensitivity of visual function testing and ERG likely results from a difference in the number of functional photoreceptors that are required for improvement in vision vs. the number required to create a large enough electrical signal to be detectable by a corneal electrode. Transgene mediated expression of *Pde6* was sufficient to slow the typically very rapid loss of photoreceptors in the *Pde6a* mutant dog. Pde6 that formed in outer segments in the treated region was able to hydrolyze cGMP as evidenced by the large reduction of cGMP detectable by IHC restricted to the retinal regions that had Pde6 expression. Improved trafficking of rhodopsin to the outer segments in treated retinal regions largely reversed the mislocalization that occurred in untreated and control treated retinal regions. Rhodopsin mislocalization is a common finding in diseased and stressed rod photoreceptors (see Hollingsworth and Gross, 2012 for a review). Structural preservation of the outer nuclear layer was detectable both on SD-OCT, where it corresponded with the topography of the treated region, and in retinal sections where it corresponded with the region of increased Pde6 expression. There also appeared to be a positive effect on rod to bipolar cell synapses. In untreated retinal regions rod bipolar cell dendritic retraction was apparent. Treatment appeared to reverse, or prevent this change.

These results show that some rescue of rod function and rod preservation is possible in this severe retinal degeneration model. Previously we had not been able to achieve rescue using AAV5 vectors and hypothesized that the faster transduction rate achieved by AAV8 vector with a capsid mutation might allow us to achieve rescue, i.e., AAV8(Y733F). The greater success with an AAV8(Y733F) construct over an AAV5 serotype delivering the same construct (Petersen-Jones, unpublished findings) is in contrast to the findings in the *Pde6b* dog, which also has a rapid photoreceptor degeneration, where results with an AAV5 vector were similar to that achieved with AAV8 (Petit et al., 2012). Conversely in rodent studies the stronger expression achieved by an AAV8 vector and an AAV8(Y733F) over AAV5 has been shown to allow for rescue of some of the more severe photoreceptor degeneration phenotypes such as the *Aipl1*^−/−^ mouse model, that has a very rapid retinal degeneration (Sun et al., 2010) and also the *Pde6b*^*rd10*^ mouse model which has a slower photoreceptor degeneration (Pang et al., 2011). We had also previously shown in a reporter gene study that an AAV8(Y733F) vector had a stronger and faster onset of reporter gene expression compared to AAV5 vectors when delivered by subretinal injection in the dog (Mowat et al., 2014).

*Pde6a* mutant dogs, in addition to lacking detectable Pde6a on Western blot analysis of pre-degenerate retinal tissue, also lack the other Pde6 subunits (Pde6b and Pde6g) (Tuntivanich et al., 2009). This is in contrast to the findings in *Pde6b* mutant dogs where the other Pde6 subunits were detectable on Western blotting of retinal tissue (Suber et al., 1993). It is not clear whether the lack of the other Pde6 subunits in *Pde6a* mutant dogs explains the greater difficulty we have faced in achieving rescue compared with the results reported for the *Pde6b* mutant dog (Petit et al., 2012; Pichard et al., 2016).

Based on these promising preliminary gene augmentation therapy results using AAV8(Y733F) to deliver *Pde6a* under control of a ubiquitous promoter further studies are warranted to optimize the degree of rescue and minimize adverse effects. This could include optimization of construct with respect to promoter to specifically target rods and possibly other modifications such as addition of an enhancer element or mini-intron. The *Pde6a* cDNA is too large to allow packaging in a self-complementary vector which could otherwise hasten the onset of expression (Petersen-Jones et al., 2009). A dose escalation study is also needed to establish the optimal therapeutic dose. It is conceivable that the variability of the degree of rescue achieved could mean that the current dose is on the edge of the most effective range, either giving too little expression, meaning that the threshold for rescue was not achieved in each eye, or too high a level of expression resulting in a range of adverse effects. With a condition with such a rapid degeneration the timing of the injection is also likely to be important and that to improve results younger animals with a larger number of surviving rods should be targeted. However, in our study more consistent results were achieved by injecting slightly older animals. Visualization in younger animals during the injection can be challenging because the ocular media is not always completely clear and remnants of development vasculature (pupillary membrane, tunica vasculosa lentis, and hyaloid vasculature) are often present. Thus, obtaining a good subretinal bleb in younger animals presents a technical challenge thus limiting the lower end of the age range that can be treated efficiently. In mouse Pde6 models, introduction of expression of the missing Pde6 subunit achieved rescue even with more advanced loss of photoreceptors (Wert et al., 2013; Koch et al., 2015). Although unlike the *Pde6a* mutant dog neither model had a complete absence of Pde6 in the pre-degenerate retina.

Limitations of this study include the necessarily small sample size due to utilization of a large animal model species. Statistical evaluation was therefore limited in many comparisons, particularly as half of the animals underwent no treatment in the contralateral control eye, the other half of the animals were injected with a control vector, more suitable to compare to the treated *AAV-Pde6a* treated eyes, as the effects of injection are controlled. We chose both untreated and control vector treated controls to allow for possible adverse effects of subretinal injection and expression of the GFP reporter gene and conversely possible trophic factor release due to the same interference. The titer of AAV-GFP used was one that would not be expected to lead to loss of photoreceptors due to high expression levels of GFP. Due to limitations of equipment availability in the laboratory at the time of the experiments, certain studies could not be performed on both cohorts of animals limiting the full evaluation of outcome of therapy. Clearly, further work is necessary to expand on these promising initial results aiming to optimize treatment efficacy, and future experiments would consider these factors.

The gene augmentation therapy in this study was not without complications. Adverse retinal changes occurred with both the *AAV-Pde6a* and the *AAV-GFP* vectors. In some eyes this resulted in focal lesions of severe retinal thinning within the injected region. These degenerate lesions occurred directly adjacent to other retinal areas where transgene expression was present and where in *AAV-Pde6a* injected eyes but not the *AAV-GFP* injected eyes there was retinal preservation. Retinal folds and rosettes were also apparent in some eyes. Retinal lesions and inflammation following subretinal AAV vectors in dogs has previously been described. Bainbridge et al., reported that 3 out of 8 dogs that were treated by subretinal injection of an AAV2 construct expressing GFP under control of a CMV promoter developed delayed ocular inflammation (Bainbridge et al., 2003). Beltran et al found that some dogs developed patches of retinal degeneration and more severe inflammatory response following subretinal administration of an AAV5 expressing GFP under different promoters (Beltran et al., 2010). In our study, no signs of retinal inflammation were detected in any eye at any timepoint, either *in vivo* or histologically. In the current study dogs were treated prior to retinal maturation, which in the dog is considered to be complete at about 7–8 weeks of age (Gum et al., 1984). It is not clear if this might be a factor in rosette formation or whether that change might be associated with the retinal detachment that is induced by the subretinal injection. Similar rosettes and areas of retinal thinning were reported in a safety study of an *AAV-RPE65* vector in adult dogs. The effect was dose related, with development of lesions occurring in eyes injected with a higher vector dose (Jacobson et al., 2006). A similar gene therapy study in which *Pde6b* mutant dogs were injected at 20 days of age with AAV vectors expressing Pde6b under control of a tissue specific promoter (Rhodopsin Kinase) to target photoreceptors did not report similar rosette formation (Petit et al., 2012). In contrast, our study used a ubiquitous promoter so off target effects are a possibility, although we did not identify Pde6 protein presence outside of the rod outer segments. It is important to note that within the treated retinal regions the retina adjacent to the degenerate lesions showed Pde6 localization to the outer segments and preservation of the outer nuclear layer. The process of performing a subretinal injection causes a temporary retinal detachment which can result in deleterious effects (Cook et al., 1995; Fisher et al., 2001; Fisher and Lewis, 2003). These could potentially be even more damaging in a diseased retina. Complications potentially associated with subretinal injections have also been reported in *RPE65* clinical trials, including foveal thinning and development of a macular hole (Maguire et al., 2008; Jacobson et al., 2012).

Here we report that AAV-mediated gene augmentation in a large animal model of rapid onset arRP caused by a *Pde6a* mutation results in Pde6 expression in rod photoreceptor outer segments restores a degree of rod photoreceptor function, delays photoreceptor degeneration, and reduces rhodopsin mislocalization, GFAP activation and bipolar cell dendrite retraction. Future studies will be designed to improve the degree and duration of rescue and to avoid adverse treatment effects that were associated with the therapy. These additional studies will be important prior to translation of this early proof-of-concept functional success with the *Pde6a* mutant dog model into clinical trials for human arRP.

## Author contributions

FM: Evaluation of retinal samples and immunohistochemistry, data interpretation and wrote the paper. LO: SD-OCT analysis and some IHC, review of paper. JB: Helped in design, assisted in subretinal injections, critical review of manuscript. KG: Performed outcome measures and data analysis, reviewed manuscript. AB: Measurement of retinal sections and cells, reviewed manuscript. JQ: Assisted in all animal experiments and performed ERG and vision testing. AD: Design and production of viral vectors. SB: Design of vector approach and production of viral vectors, critical review of manuscript. WH: Design of vectors, production of viral vectors, critical review of manuscript. SP: Designed and oversaw experiments, subretinal injections, co-wrote the manuscript.

### Conflict of interest statement

WH and the University of Florida have a financial interest in the use of AAV therapies, and own equity in a company (AGTC) that might, in the future, commercialize some aspects of this work. The other authors declare that the research was conducted in the absence of any commercial or financial relationships that could be construed as a potential conflict of interest.
