# Supplementary material for: Gene Therapy in a Large Animal Model of PDE6A-Retinitis Pigmentosa
Source: Front Neurosci. 2017 Jun 20;11:342. doi: 10.3389/fnins.2017.00342 (PMC5476745; doi:10.3389/fnins.2017.00342)
Supplement: Supplementary file 2 [file Image1.PDF]

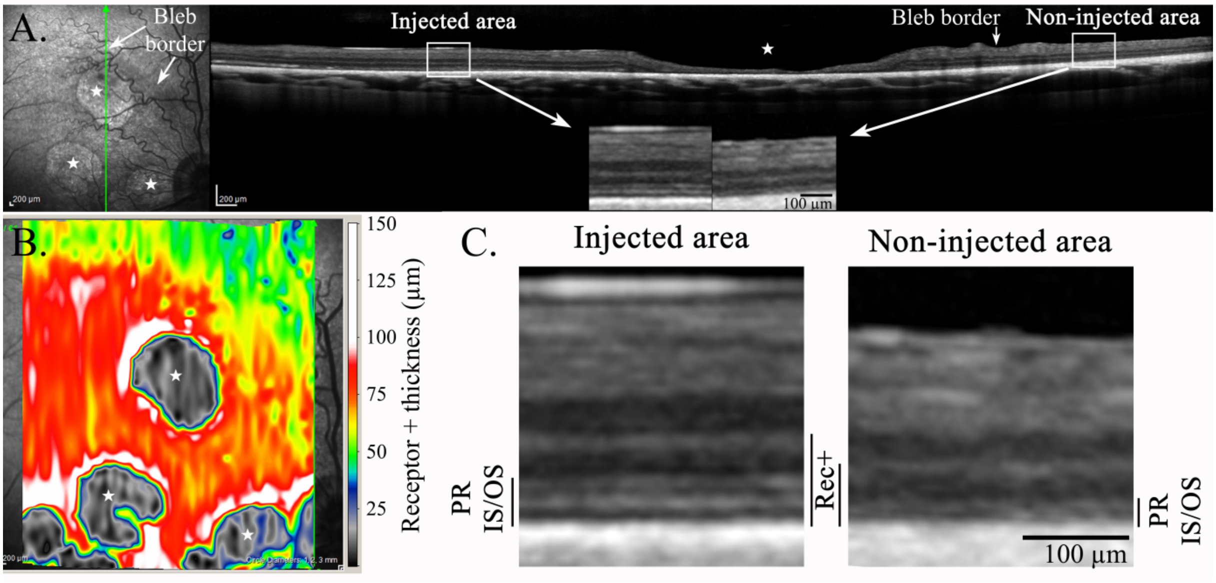


**Supplementary Figure 1. Gene therapy shows evidence of preservation of photoreceptor thickness and morphology in the treated area on SD-OCT images.** Images were captured 5 months following injection in an affected animal (12-047) treated at 41 days of age. **A.** cSLO (image on the left) and corresponding SD-OCT cross sections image (image on the right) showed the border between the treated and untreated regions (white arrows). The SD-OCT retinal cross-section corresponds to the area shown by the green arrow on the cSLO image. The SD-OCT image shows preservation of the retinal thickness in the injected (treated) area versus in the non-injected area. **B.** Receptor plus (Rec+) heat map thickness showing the thicker photoreceptor layers in the treated area (red) versus untreated area (green – top right of the image). Rec+ is a representative measure of the total length of the photoreceptor (from the outer segment to the outer plexiform layer). **C.** Magnified images of an injected (treated) area and non-injected (untreated) area from the cross section in **A.** This shows the total retina thickness preservation in the treated region but more importantly the photoreceptor inner/outer segments (PR IS/OS) thickness and morphology preservation. PR IS/OS normal morphology in the treated area can be seen as clearly defined reflective (white) and non-reflective (dark) zones/bands only in the injected area. In the non-injected area these zones cannot be discerned indicating shortening of the PR IS/OS.

Areas of degeneration indicated by the white stars can be seen in **A.** and **B**.
